# Vancomycin pharmacokinetics in critically ill obese patients: can the clinician sit back and relax?

**DOI:** 10.1186/s13054-019-2311-2

**Published:** 2019-01-17

**Authors:** Patrick M. Honore, David De Bels, Luc Kugener, Sebastien Redant, Rachid Attou, Andrea Gallerani, Herbert D. Spapen

**Affiliations:** 1 ICU Department, Centre Hospitalier Universitaire Brugmann-Brugmann University Hospital, Place Van Gehuchtenplein, 4, 1020 Brussels, Belgium; 2 Medicine, Ageing & Pathology Research Group, Vrije Universiteit Brussel, Brussels, Belgium

The recently published study by Lin et al. provides valid pharmacokinetic (PK) data regarding continuous infusion of vancomycin (CIV) in obese versus non-obese patients. An important finding is that CIV in obese patients, whether or not receiving renal replacement therapy, consistently produced target “therapeutic” serum concentrations and resulted in a lower weight-based daily vancomycin exposure as compared to non-obese subjects [1].

A shortcoming of this study is the lack of information on bacterial susceptibility to vancomycin. Vancomycin achieves a near maximal bactericidal effect when the ratio of the vancomycin area under the concentration-time curve (AUC) over the minimum inhibitory concentration (MIC) exceeds 400 [2]. However, vancomycin exerts slow bactericidal activity and has low tissue penetration, and serum levels poorly correlate with microbiological or clinical success. Moreover, high vancomycin MICs, irrespective of testing methodology and infection source, are predictive for treatment failure and associated with a higher mortality rate [3]. Vancomycin trough concentrations of 15 mg/L (with intermittent administration) or steady state concentrations of 20 to 30 mg/L (with CIV) act as surrogates of an AUC/MIC ≥ 400, assuming a vancomycin MIC of ≤ 1 mg/L. The majority of cultured bacteria in the study of Lin et al. are coagulase-negative staphylococci which remain largely susceptible to vancomycin in adult patients [4]. Less than 10% of cultures grew methicillin-resistant *Staphylococcus aureus* (MRSA). Vancomycin may not be useful for treating serious MRSA infections with MIC values > 1 mg/L, and no dosing regimen can reach an AUC/MIC for isolates with a vancomycin MIC ≥ 2 mg/L [3].

Lin et al. also did not assess vancomycin concentrations after the loading dose (approximately 25 mg/kg) and at 24 h. In septic patients, Cristallini et al. [5] applied a loading dose of 35 mg/kg followed by a daily CIV dose adapted to creatinine clearance. Therapeutic concentrations of 20 to 30 mg/L were obtained in 54% of patients after 24 h. Thus, early relevant vancomycin levels were obtained in only half of a representative critically ill patient cohort despite utilizing a substantially higher loading dose and aiming at higher steady state vancomycin concentrations than Lin et al.

From a PK viewpoint, the study of Lin et al. is highly informative, yet some restraint is required before translating these results into clinical scenarios.

## Abbreviations

AUC: Area under the concentration-time curve
CIV: Continuous infusion of vancomycin
MIC: Minimum inhibitory concentration
MRSA: Methicillin-resistant *Staphylococcus aureus*
PK: Pharmacokinetic
